# Characterization of Distinct CyanoHABs-Related Modules in Microbial Recurrent Association Network

**DOI:** 10.3389/fmicb.2019.01637

**Published:** 2019-07-17

**Authors:** Seong-Jun Chun, Yingshun Cui, Chang Soo Lee, A Ra Cho, Kiwoon Baek, Ahyoung Choi, So-Ra Ko, Hyung-Gwan Lee, Seungwoo Hwang, Hee-Mock Oh, Chi-Yong Ahn

**Affiliations:** ^1^Cell Factory Research Center, Korea Research Institute of Bioscience and Biotechnology (KRIBB), Daejeon, South Korea; ^2^Department of Environmental Biotechnology, KRIBB School of Biotechnology – Korea University of Science and Technology (UST), Daejeon, South Korea; ^3^Division of Freshwater Bioresources Research, Nakdonggang National Institute of Biological Resources, Sangju, South Korea; ^4^Division of Freshwater Bioresources Culture Research, Nakdonggang National Institute of Biological Resources, Sangju, South Korea; ^5^Korean Bioinformation Center (KOBIC), Korea Research Institute of Bioscience and Biotechnology (KRIBB), Daejeon, South Korea

**Keywords:** cyanoHABs, bacterial community, microbial recurrent association network, cyanoHABs-related module, connector, module hub

## Abstract

To elucidate the interspecies connectivity between cyanobacteria and other bacteria (non-cyanobacteria) during cyanobacterial harmful algal blooms (cyanoHABs), samples were collected from the Nakdong River, Korea, from June 2016 to August 2017, and microbial recurrent association network (MRAN) analysis was performed to overcome the limitations of conventional network analysis. *Microcystis* blooms were tightly linked with *Pseudanabaena* in summer and were accompanied by significant changes in the non-cyanobacterial community composition (nCCC) compared to non-bloom period. Riverine bacterial communities could be clearly separated into modules that were involved in the formation, maintenance, and decomposition of cyanoHABs. *Roseomonas* and *Herbaspirillum* were directly linked with major cyanobacteria and assigned to connector and module hub in cyanoHABs-related modules, respectively. The functional profiles of the cyanoHABs-related modules suggested that nitrate reduction, aerobic ammonia oxidation, fermentation, and hydrocarbon degradation could be increased during the *Microcystis* bloom periods. In conclusion, MRAN analysis revealed that specific bacteria belonging to cyanoHABs-related module, including connectors and module hubs, appeared to contribute to the development and collapse of cyanoHABs. Therefore, to understand cyanoHABs, a modular microbial perspective may be more helpful than a single bacterial species perspective.

## Introduction

In freshwater ecosystems, eutrophication and subsequent algal proliferation is one of the most important environmental issues (Heisler et al., 2008). There is particular concern regarding the proliferation of cyanobacteria, such as *Microcystis, Dolichospermum* (formerly *Anabaena*), and *Aphanizomenon*, which form cyanobacterial harmful algal blooms (cyanoHABs); these cyanoHABs cause serious problems regarding public health, water management, and remediation (Carmichael, 2001; Paerl and Otten, 2013; Rastogi et al., 2015). Previous studies have focused on the relationship between algal bloom formation and environmental parameters and found that environmental variables (e.g., temperature, nutrients, and light) play an important role in the formation of blooms. However, the species that cause cyanoHABs do not live alone in aquatic ecosystems; rather, these species consistently interact with various microorganisms in a variety of ways, ranging from predator-prey interactions to mutualistic interactions.

Microorganisms consist of viruses, bacteria, archaea, and protists, and they generate diverse ecological relationships that form a complex ecosystem (Coyte et al., 2015). Through these interactions, microorganisms can overcome their weakness and enhance their strengths (Faust and Raes, 2012). Recently, many studies have focused on microbial interactions during cyanoHABs and revealed that heterotrophic bacteria and cyanobacteria are closely linked with each other during bloom periods (Louati et al., 2015; Xie et al., 2016; Woodhouse et al., 2018). Furthermore, Tromas et al. (2017) found that bacterial community data predicted cyanoHABs more accurately than conventional prediction model based on water quality and environmental variables. Cyanobacteria can provide a favorable environment for heterotrophic bacteria by producing extracellular polysaccharides (EPS), oxygen, and cyanobacteria-derived substances (Rossi and De Philippis, 2015). Heterotrophic bacteria can produce bacteria-derived substances, such as nutrients and microelements, for cyanobacteria by decomposing organic polymers into small molecules that can be readily used by cyanobacteria (Paerl, 1974; Wang et al., 2016). These interactions could play an important role in algal proliferation and decomposition processes in aquatic ecosystems. Therefore, clarifying the microbial interactions is a crucial step for better understanding of cyanoHABs.

Microbial community data that are generated by next-generation sequencing can provide information on the relative abundances of thousands of different microorganisms in a specific environment (Klindworth et al., 2013). With an increasing amount of information and advanced computational techniques, biological association networks are becoming a fundamental tool for the investigation of high-throughput data in biology (Faust and Raes, 2012; Röttjers and Faust, 2018). The power and usefulness of biological association networks originate from their ability to extract new information on ecological interactions, organizations, keystone organisms (i.e., hubs), and their responses to environmental variables that could not be revealed by conventional techniques. Recently, association network analyses have been applied to investigate a broad range of microbial interactions in various environments, including soil, marine, and freshwater environments (Barberán et al., 2012; Eiler et al., 2012; Chow et al., 2014). Network analysis has been applied in several studies and discovered that the species-specific associations between cyanobacteria and bacterioplankton can provide crucial information regarding bloom formation (Woodhouse et al., 2016, 2018; Yang et al., 2017).

To explore the microbial interactions underlying cyanoHABs in a river microbiome, we collected samples from the Nakdong River, Korea, from June 2016 to August 2017; serious cyanobacterial blooms have been observed every year in the Nakdong River (Hur et al., 2013; Srivastava et al., 2015; Hong et al., 2018). The Nakdong River is the second largest river system in Korea, and this river supplies drinking water to over 10 million people and is also used for agricultural and industrial purposes. We employed high-throughput sequencing of the 16S rRNA gene to determine the composition of the *Cyanobacteria* and non-cyanobacterial communities and assess how these communities were impacted by cyanoHABs. Analysis of a microbial recurrent association network (MRAN) was performed to understand the interspecies connectivity and identify the ecological roles of individual species. Furthermore, functional profiles were constructed. Finally, by exploring the repetitive dynamics of the river community, we addressed the following questions: how does the non-cyanobacterial community composition (nCCC) vary with different cyanoHABs? Are there any modules with patterns associated with cyanoHABs? Which bacteria are directly linked with harmful cyanobacteria? Finally, what role do these bacteria have in the microbial network?

## Materials and Methods

### Sample Collection and Water Quality Analysis

Freshwater samples were collected monthly from June 2016 to August 2017 at depths of 0 and 2 m from three sites (MG1, MG2, and USF) on the Nakdong River, Korea (for a total of 69 samples) (Supplementary Figure S1). Water quality parameters [temperature, pH, and dissolved oxygen (DO)] were measured using a portable instrument (YSI ProDSS Multiparameter, Yellow Springs, OH, United States). Total nitrogen (TN), total dissolved nitrogen (TDN), total phosphorus (TP), and total dissolved phosphorus (TDP) were determined according to the Standard Methods for the Examination of Water and Wastewater (American Public Health Association [APHA] et al., 2005). The chlorophyll-*a* (chl-*a*) concentration was calculated using the method of Lorenzen with a spectrophotometer (Thermo Fisher Scientific, Vantaa, Finland) (Lorenzen, 1967). Monthly precipitation and discharge data were obtained from the Korea Meteorological Administration and the National Institute of Environmental Research (NIER), respectively.

### Cyanobacteria and Non-cyanobacterial Community Composition

For the bacterial community analysis, 1 L water samples were collected and stored in a cooler until arrival (within 2 h) at the Korea Research Institute of Bioscience and Biotechnology (Daejeon, South Korea); filtering began immediately upon sample arrival at the facility. Freshwater samples were filtered using a sterilized 0.22-μm polycarbonate membrane filter (Millipore Corporation, Bedford, MA, United States). All membrane filters were stored at -80°C in a deep freezer until DNA extraction. Genomic DNA was extracted using a ChargeSwitch^®^Forensic DNA Purification Kit (Invitrogen, Carlsbad, CA, United States) according to the manufacturer’s instructions. The bacterial 16S rRNA gene was amplified using a universal bacterial primer set, 341F/805R (341F: CCTACGGGNGGCWGCAG; 805R: GACTACHVGGGTATCTAATCC), which targets the V3–V4 regions (Herlemann et al., 2011). PCR amplification was performed using Ex Taq^TM^ Hot Start Version (Takara Bio Inc., Otsu, Japan). The average read length was approximately 400 bp after trimming the barcode and primer sequences. The obtained amplicons were then purified using Agencourt AMPure XP beads (Beckman Coulter, Brea, CA, United States) according to the manufacturer’s instructions. The quantification of the DNA concentration was performed using a Quant-iT dsDNA HS Assay Kit (Thermo Fisher Scientific, Waltham, MA, United States); then, the purified amplicons were pooled in equimolar concentrations. The MiSeq reagent kit v3 (Illumina) was used for the long paired-end reads (2 × 300 bp) sequencing reactions, and samples were sequenced using a high-throughput paired-end Illumina sequencer (MiSeq, 2 × 250 bp reads) from the Macrogen Corporation (Seoul, South Korea).

The resulting sequences were processed using mothur (Schloss et al., 2009) according to the MiSeq standard operating procedure^1^ (Kozich et al., 2013). Briefly, low-quality sequences were removed from the analysis if they contained ambiguous characters, contained more than two mismatches to the forward primer or one mismatch to the barcode, or were less than 300 bp or more than 500 bp in length. After removing singletons, doubletons, and tripletons, the pre-cluster method was applied to further reduce any sequencing errors produced by the MiSeq Illumina sequencing platform. Chimeras were identified and removed using the chimera.uchime command (Edgar et al., 2011). The Silva database (release 123) was used to align and classify the sequences. A similarity cut-off of >99% was used to assign the same operational taxonomic units (OTUs). In addition, we split the 16S rRNA gene sequence data sets into two partitions, the “cyanobacterial community” and “non-cyanobacterial community.” The bacterial 16S rRNA gene sequences and accompanying metadata have been deposited in the Sequence Read Archive (SRA) of NCBI under the project number PRJNA479553.

Blooms can be defined in numerous ways. In this study, we focused on *Microcystis* blooms and defined cyanobacterial bloom when the proportion of specific cyanobacterial OTU exceeds 10% of total bacterial composition and chl-*a* exceeds 15 μg/L (Ahn et al., 2007). *Aphanizomenon* OTU exceeded 80% of total bacterial composition in winter samples, although chl-*a* concentration was below 15 μg/L. We also added this period as an exceptional case of bloom period.

The abundance of cyanobacterial 16S rDNA copies was determined using SYBR^®^Premix Ex Taq^TM^ II (Takara Bio Inc., Otsu, Japan) according to the manufacturer’s protocol. To standardize the target gene quantities, single copy insert plasmids were used to establish standard curves. Real-time quantitative PCR (qPCR) reactions were then conducted at 95°C for 30 s, followed by 40 cycles of 95°C for 5 s and 55°C for 30 s in the BioRad Chromo4 real-time PCR system (Bio-Rad, Hercules, CA, United States). The primer sequences were as follows: CYAN108F/16SCYR (CYAN108F: ACGGGTGAGTAACRCGTRA and 16SCYR: CTTCAYGYAGGCGAGTTGCAGC) (Rinta-Kanto et al., 2005). The phylogenetic tree of cyanobacterial OTUs was constructed using a neighbor-joining (Saitou and Nei, 1987) algorithm in MEGA 6 (Tamura et al., 2013), and bootstrap values were calculated from 1000 replicates. The 16S rRNA gene sequences for the reference species were selected by searching the closest relatives in the arb database and were retrieved from the NCBI public database. The richness (Chao1) and diversity (Shannon’s index) indices were calculated after subsampling using the Mothur software package (Schloss et al., 2009).

### MRAN, Topological Features, and Different Topological Roles of Individual Nodes

To understand the relationship among cyanobacteria, non-cyanobacterial community members, and environmental variables, a total of six association networks (three sites and two depths) were constructed by extended local similarity analysis (eLSA) using a linear interpolation with significant correlations (*P* < 0.001) (Xia et al., 2011). Only the correlations that appeared continuously for more than 6 months were used in the further study. An association network visualizes co-occurrence patterns among OTUs and allows us to identify cyanobacteria-related bacterial OTUs. Because network analysis is a correlation-based technique, the correlations do not always reflect the complex and dynamic natural microbial interactions that exist in a real environment (Faust and Raes, 2012). To overcome this weakness, the MRAN, which is a consensus network extracted from the recurrent associations that were observed at multiple sites and depths, was built. The MRAN was extracted using correlations that were repeated three or more times among the six association networks described above. The network was visualized using the open source software Cytoscape 3.5.1 (Shannon et al., 2003). Random undirected networks of equal size in terms of the number of nodes and edges were calculated by the Erdős–Rényi model using the Network Randomizer plugin in Cytoscape. The network topological features, including the clustering coefficient (*C*), characteristic path length (*L*), centralization, network density, diameter, and modularity analysis (modules were identified using the Louvain algorithm), were calculated using the Network Analyzer plugin in Cytoscape and R software (package: igraph) (Csardi and Nepusz, 2006; Assenov et al., 2007).

The topological parameters were as follows: (1) the clustering coefficient (*C*), which represents the average fraction of pairs of species one link away from a species that is also linked to another; (2) the characteristic path length (*L*), which is the average shortest path between all pairs of species (Watts and Strogatz, 1998; Albert and Barabási, 2002); and (3) the centralization, which is the degree of dispersion of all node centrality scores in a network from the maximum centrality score obtained in the network. Furthermore, we calculated the small-world coefficient (SW), in which higher values indicate a greater small-worldliness of the network (Davis et al., 2003). The SW is defined as SW = (*C*/*C_R_*)/(*L*/*L_R_*) (*R* represents the parameters from a random network). The average monthly patterns of major modules were calculated using the normalized (feature scaling) relative abundances of non-cyanobacterial OTUs. To identify the topological roles of each node in the microbial network, we calculated the within-module connectivity (*Z_i_*: used to describe how well a node is connected to other nodes in the same module) and the among-module connectivity (*P_i_*: used to describe how well a node is connected to the nodes in other modules) (Guimera and Amaral, 2005). According to the threshold values of *Z_i_* and *P_i_* described in Olesen et al. (2007), the whole network was divided into four categories: (1) peripherals (*Z_i_* ≤ 2.5, *P_i_* ≤ 0.62), which refer to nodes that do not interact much with other nodes within and among the modules; (2) module hubs (*Z_i_* > 2.5, *P_i_* ≤ 0.62), which refer to nodes that have many interactions with other nodes within the module; (3) connectors (*Z_i_* ≤ 2.5, *P_i_* > 0.62), which refer to nodes that have many interactions with other nodes among the modules; and (4) network hubs (*Z_i_* > 2.5, *P_i_* > 0.62), which refer to nodes that act as both module hubs and connectors.

### Functional Annotation of Modules and Statistical Analysis

To investigate the functional annotation of major modules, “functional annotation of prokaryotic taxa” (FAPROTAX) was performed using python collapse_table.py with the normalized OTU table for each module (Louca et al., 2016). The FAPROTAX dataset is available at http://www.zoology.ubc.ca/louca/FAPROTAX. FAPROTAX is a manually constructed database based on the literature on cultured representatives that focuses on marine and freshwater microbiomes. The functional group abundances in each module were calculated by multiplying the calculated values (“function tables”) by the total sum of the OTUs belonging to each major module. A heatmap illustrating the FAPROTAX data was generated using the “heatmap.2” function in gplot.

All statistical analyses were performed using the R software environment (version 3.4.0). The hierarchical clustering analysis was based on the Bray–Curtis distance. To determine the relationships among the environmental variables, major cyanobacterial OTUs and non-cyanobacterial communities, distance-based redundancy analysis (db-RDA) using a Bray-Curtis distance matrix was performed for constrained ordination using the “capscale” function in vegan package (Oksanen et al., 2013). To investigate the differences in major modules, a non-metric multidimensional scaling (NMDS) analysis was conducted on the top 50 genera using the “metaMDS” function in vegan package. The statistical significance of the nCCC between sampling times was analyzed using PERMANOVA, which analyses multivariate data on the basis of any measure of distance using permutations with the “adonis” function in the vegan package. All permutational tests were conducted with 999 permutations. Student’s *t*-test was used for statistical comparisons of the relative abundances of the non-cyanobacterial OTUs (at the family level).

## Results

### Cyanobacterial Community Composition

A total of 1,920,658 high-quality sequences remained after processing, and 6993 OTUs were identified at a 99% similarity cut-off. These OTUs belonged to 41 phyla, 95 classes, 182 orders, 357 families, and 700 genera. Among these OTUs, 6785 OTUs were assigned to non-cyanobacterial community members, while 208 OTUs represented *Cyanobacteria*.

**FIGURE 1:**
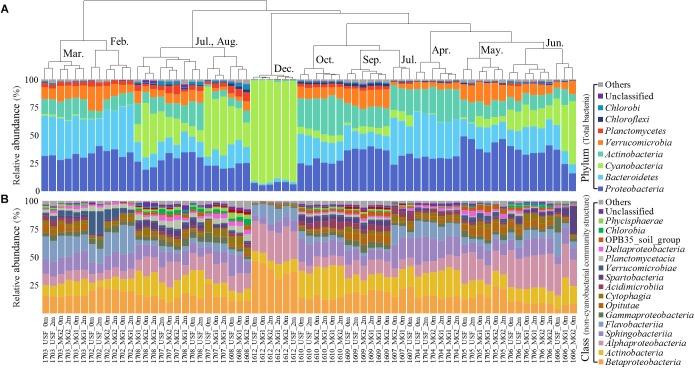
Relative abundance of **(A)** total bacterial community structure (phylum level) and **(B)** non-cyanobacterial community structure (class level). The dendrogram shows the clustering of the OTU data (Bray–Curtis similarity measure). Taxa with <0.5% average abundances are pooled together and shown as “Others.” Samples are labeled by a four-number code indicating the year and month of sampling (in YYMM format).

**FIGURE 2:**
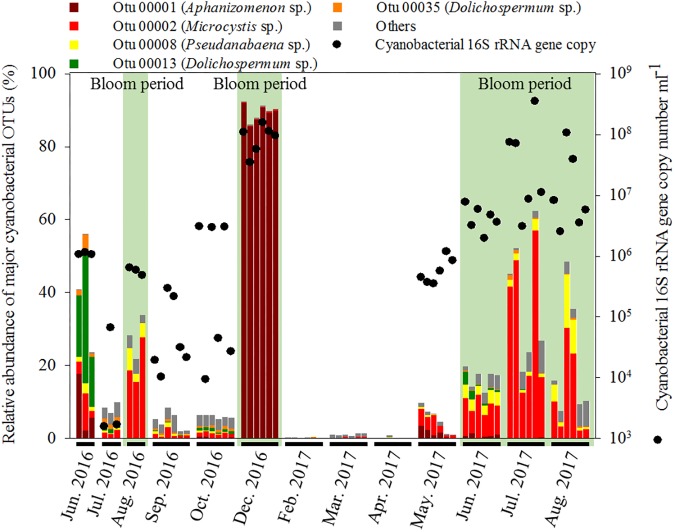
Relative abundance of major cyanobacterial OTUs in the bacterial community and 16S rRNA gene copy number of *Cyanobacteria*.

The hierarchical clustering analysis based on the Bray–Curtis distance showed that communities within the same month tended to cluster together (Figure 1). The relative abundance of *Cyanobacteria* varied over the sampling stations and period, ranging from 0.29 ± 0.14% to 89.69 ± 2.31% of the total bacterial community. To classify the major cyanobacterial OTUs, the 10 most abundant OTUs were used to construct a phylogenetic tree with the reference sequences (Supplementary Figure S2). The major cyanobacterial OTUs were OTU00001, OTU00002, OTU00008, OTU00013, and OTU00035 (Figure 2), which were identified as *Aphanizomenon, Microcystis, Pseudanabaena*, and *Dolichospermum* (2 OTUs, formerly *Anabaena*), respectively. *Microcystis* and *Aphanizomenon* were the most abundant cyanobacterial OTUs during the investigation period. *Microcystis* blooms were observed in August 2016 and persisted from June to August 2017; however, the sole *Aphanizomenon* bloom was observed only temporarily in December 2016. *Aphanizomenon* also showed a temporary small increase in June 2016 and May 2017 before a *Microcystis* bloom. Interestingly, *Pseudanabaena* appeared simultaneously with *Microcystis* (*R* = 0.63, *P* < 0.001), and two *Dolichospermum* OTUs co-occurred in June 2016. To quantify cyanobacterial abundance, cyanobacterial 16S rRNA copy numbers were determined using qPCR. During the *Microcystis* and *Aphanizomenon* bloom periods, abundances increased to 3.5 × 10^8^ copies ml^-1^ in July 2017 and 1.6 × 10^8^ copies ml^-1^ in December 2016, respectively. The relative abundance of *Microcystis* and the copy numbers were significantly correlated during June-August in 2016 and 2017 (*R* = 0.87, *P* < 0.001).

### Non-cyanobacterial Community Composition

The non-cyanobacterial community changed significantly during the outbreak and decline of the cyanoHABs. *Proteobacteria* (39.52 ± 11.19%) (mainly *Betaproteobacteria* and *Alphaproteobacteria*), *Bacteroidetes* (25.30 ± 8.61%) (mainly *Sphingobacteriia* and *Flavobacteriia*), and *Actinobacteria* (17.60 ± 7.21%) dominated the nCCC during the sampling period (Figure 1). At the family level, the relative abundances of *Acetobacteraceae, Alcaligenaceae, Cytophagaceae*, OPB56, *Planctomycetaceae, Phycisphaeraceae*, and *Xanthomonadaceae* in the nCCC were significantly higher during *Microcystis* bloom periods than during non-bloom periods (*P* < 0.001) (Supplementary Figure S3).

**FIGURE 3:**
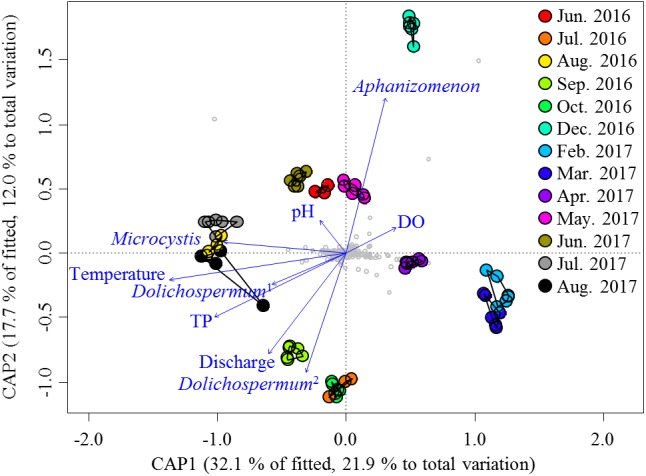
db-RDA ordinations of the non-cyanobacterial community based on the Bray–Curtis distance. Vectors indicate the direction of the significant parameter (*P* < 0.001) effect in the ordination plot. Small gray circles represent OTUs. *Dolichospermum*^1^ indicates OTU00013, and *Dolichospermum*^2^ indicates OTU00035.

Given the large variation in the relative abundances of the nCCC across samples, the environmental variables (Supplementary Table S1) were the potential drivers that reshaped the nCCC. PERMANOVA revealed a significant difference between bloom periods and non-bloom periods (pseudo-*F* = 11.72, *P* < 0.001). As such, the db-RDA was constructed to partition the variation in the beta diversity of the non-cyanobacterial community into fractions based on the environmental variables and cyanobacterial OTUs (Figure 3). The eigenvalues of the first and second axes were 4.10 and 2.26, respectively. The Monte Carlo permutation test was significant for all canonical axes together (pseudo-*F* = 5.51, *P* < 0.001). The db-RDA showed that approximately 34% of the total variance was explained by the constrained matrix, and it clearly segregated the pre-bloom (June 2016 and May–June 2017), bloom (August 2016 and July–August 2017), and post-bloom (September–October 2016) periods of *Microcystis*. The first axis (db-RDA1, *F* = 33.64, *P* < 0.001) was strongly related to *Microcystis* (pseudo-*F* = 4.17, *P* < 0.005), water temperature (pseudo-*F* = 29.94, *P* < 0.001) and TP (pseudo-*F* = 6.78, *P* < 0.001), while the second axis (db-RDA2, pseudo-*F* = 18.51, *P* < 0.001) was correlated with *Aphanizomenon* (pseudo-*F* = 15.27, *P* < 0.001) and discharge (pseudo-*F* = 6.37, *P* < 0.001).

**Table 1:** Topological features and statistics of the microbial association networks.

Features	Association networks^∗^ (*n* = 6)	MRAN (≥3/6)
Number of nodes	423–638	362
Number of edges	3198–9792	2072
Diameter	8–11	12
Ratio of positive/negative edge	0.86–0.92	0.92
Average number of neighbors	15.12–30.7	11.45
Network density	0.036–0.051	0.032
Network heterogeneity	0.826–1.067	1.127
Network heterogeneity, random	0.176–0.252	0.290
Centralization	0.111–0.187	0.13
Centralization, random	0.023–0.031	0.032
Modularity	0.462–0.539	0.473
Modularity, random	0.238–0.285	0.267
Average clustering coefficient (*C*)	0.403–0.477	0.318
Clustering coefficient, random (*C*_r_)	0.037–0.052	0.030
Characteristic path length (*L*)	3.03–3.95	3.53
Characteristic path length, random (*L*_r_)	2.17–2.53	2.67
*C*/*C*_r_	8.4–11.38	10.6
*L*/*L*_r_	1.36–1.67	1.32
Small-world coefficient (SW)	5.25–7.29	8.03


*^∗^Association networks for the six locations (three sites with two depths) constructed by eLSA (Supplementary Figure S4).*

### The Modular Structure and Different Topological Roles of Individual Nodes

**FIGURE 4:**
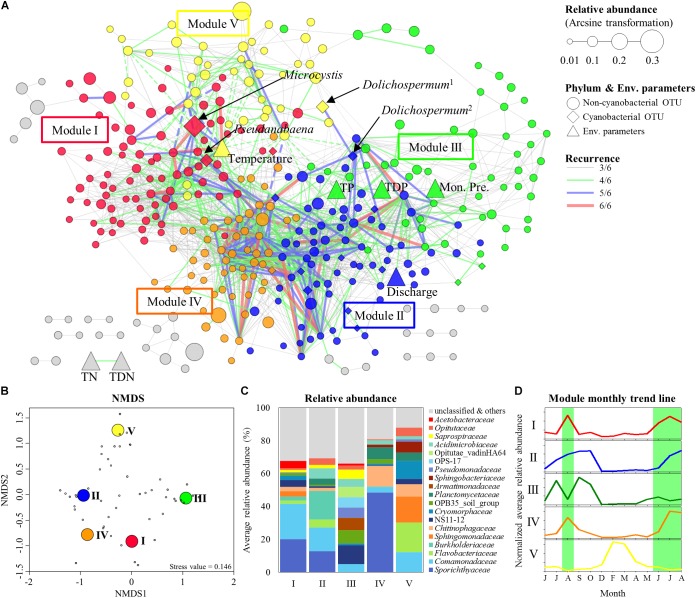
Modular structure of MRAN, comparison of nCCCs, and monthly patterns of the modules. **(A)** MRAN (≥3/6): node colors represent modules, *Dolichospermum*^1^ indicates OTU00013, *Dolichospermum*^2^ indicates OTU00035, solid lines represent positive correlations and dashed lines represent negative correlations. **(B)** NMDS of the top 50 genera in the major modules. **(C)** Average relative abundances of non-cyanobacterial OTUs in the major modules. **(D)** Trend lines representing the normalized average relative abundance of each major module: the green background represents *Microcystis* bloom periods.

To build the MRAN, 659 bacterial OTUs, 29 cyanobacterial OTUs, and 11 environmental variables were selected based on occurrence in at least 20% of the 69 total samples. Six networks (three sites with two depths) were constructed with 428–634 nodes and 3198–9792 edges using eLSA (Supplementary Figure S4). After testing for significance by a permutation test and screening by *P*-values, significant correlations were found between OTUs and environmental variables. These networks were merged into one network only when there were repeated correlations (i.e., at least three among the six networks, ≥3/6), and this merged network was called the MRAN (Figure 4A). Overall, in the MRAN, approximately 2% of the edges were repeated six times in six different networks (Supplementary Figure S4), while 70% of the edges were repeated three times.

Table 1 summarizes the topological features of the networks. The MRAN comprised 362 nodes and 2072 edges with an average of 11.45 neighbors and a characteristic path length of 3.53. The network had an overall diameter of 12 edges and an average clustering coefficient of 0.318. The average clustering coefficient and characteristic path length values were higher than the values of their Erdős–Rényi random networks. In addition, the SW value was greater than 1, suggesting that the MRAN had small-world properties. The modularity value (0.473) was higher than that of the random network (0.267). A total of 17 modules were generated, and 5 modules (I, II, III, IV, and V) were defined as major modules (occupied 89% of all nodes) (Figure 4A). *Microcystis* and *Pseudanabaena* belonged to Module I, while *Aphanizomenon* was not found in any of the MRAN modules. In addition, the specific components and average relative abundances of the nCCC were different among the major modules (Figure 4B,C). *Comamonadaceae* was the major bacterial order in Module I (21.5%), while *Sporichthyaceae* was the major bacterial order in Module IV (48.5%). There were clear differences in the monthly patterns of the major modules (Figure 4D). Modules I and IV increased and decreased with the occurrence of *Microcystis* blooms, while Module III showed the opposite pattern during bloom periods. Module II increased gradually throughout the summer, peaked in the autumn, and decreased in the winter, while Module V showed an increasing trend in the winter.

Many significant correlations were observed among the five major non-cyanobacterial phyla, *Cyanobacteria* and environmental variables (Supplementary Table S2). Specifically, 189 significant correlations were found between *Cyanobacteria* and non-cyanobacterial community members, and approximately 40% of the correlations were constructed with *Proteobacteria*. However, *Cyanobacteria* showed high connections with *Actinobacteria* (5.4% of all possible edges) and other *Cyanobacteria* (33.3%).

**FIGURE 5:**
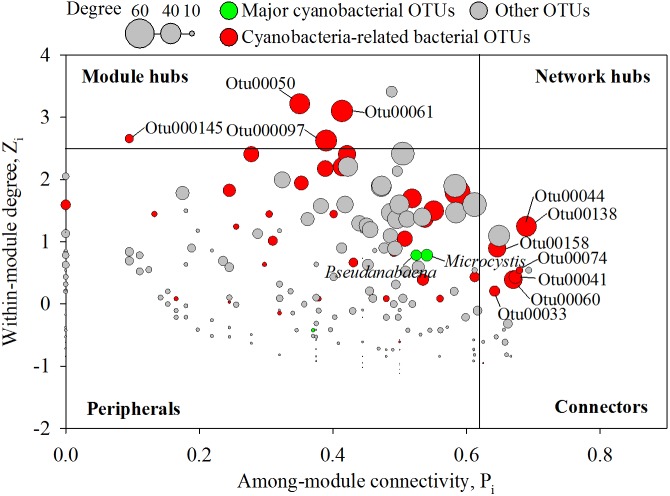
*Z_i_*–*P_i_* plot showing the distribution of nodes based on their topological roles in the networks. Colors represent cyanobacterial OTUs and cyanobacteria-related bacterial OTUs. The threshold values of *Z_i_* and *P_i_* for categorizing OTUs were 2.5 and 0.62, respectively, as simplified by Olesen et al. (2007).

**FIGURE 6:**
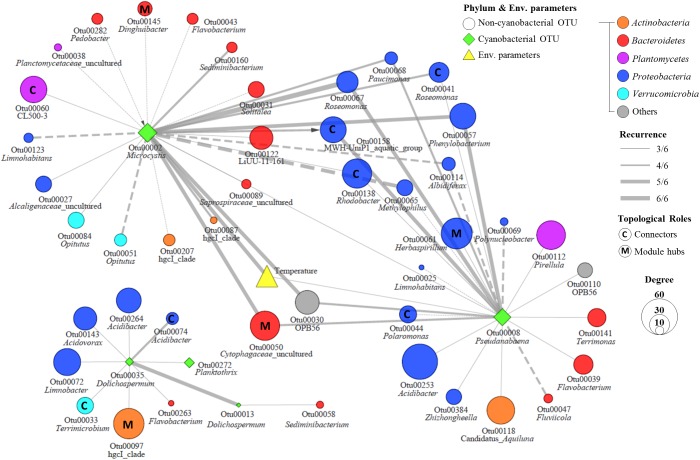
The subnetwork of major cyanobacterial OTUs in the MRAN. Node size represents node degree, solid lines represent positive correlations, dashed lines represent negative correlations, and the arrow represents a delayed correlation.

In an ecological network, the roles of individual nodes reflect the potential importance of OTUs in the microbial community. In a *Z_i_*–*P_i_* plot, all nodes fell into three categories (Figure 5). Most (93.4%) of the nodes were peripheral nodes, while only 1.4 and 5.2% of the nodes were module hubs and connectors, respectively. Among the peripherals, 46% had no links outside their own modules (i.e., *P_i_* = 0). To explore the detailed relationship and roles between the major cyanobacteria and nCCC, a subnetwork was extracted from merged network (Figure 4A) with the major cyanobacterial OTUs (Figure 6). This subnetwork comprised 51 nodes (40 peripheral nodes, 7 connectors, and 4 module hubs) and 58 edges (42 positive correlations and 16 negative correlations). *Betaproteobacteria* (29%) and *Sphingobacteriia* (17%) were the major cyanobacteria-related non-cyanobacterial classes (Supplementary Table S3). Four *Actinobacteria* OTUs, i.e., the hgcI clade (3 OTUs) and Candidatus *Aquiluna*, were also observed in subnetwork. *Microcystis* and *Pseudanabaena* were connected with each other and shared 10 non-cyanobacterial OTUs. In contrast, two *Dolichospermum* OTUs formed a relatively small network that was isolated from the main cyanobacterial network and did not share any OTUs. *Microcystis* was correlated with temperature and 25 non-cyanobacterial OTUs, including *Sphingobacteriia* (6 OTUs), *Betaproteobacteria* (6 OTUs), and *Alphaproteobacteria* (4 OTUs), with two delayed correlations. Among these OTUs, the proportions of module hubs and connectors were higher (8 and 16%, respectively) than the average proportions (4.5 and 6.8%, respectively). *Pseudanabaena* was correlated with 22 bacterial OTUs, in which 2 OTUs were module hubs (9%) and 4 OTUs were connectors (18%). Six *Proteobacteria* OTUs, i.e., one closely related to *Phenylobacterium*, MWH-UniP1 group, *Paucimonas, Rhodobacter*, and *Roseomonas* (2 OTUs), were positively correlated with both *Microcystis* and *Pseudanabaena.* OTU00041 (*Roseomonas*), which was assigned as a connector, linked *Microcystis* and *Pseudanabaena* to Module II and Module IV. OTU00050 (uncultured *Cytophagaceae*) was identified as a module hub in Module I and was connected to both *Microcystis* and *Pseudanabaena*.

### Functional Annotation for Modules

**FIGURE 7:**
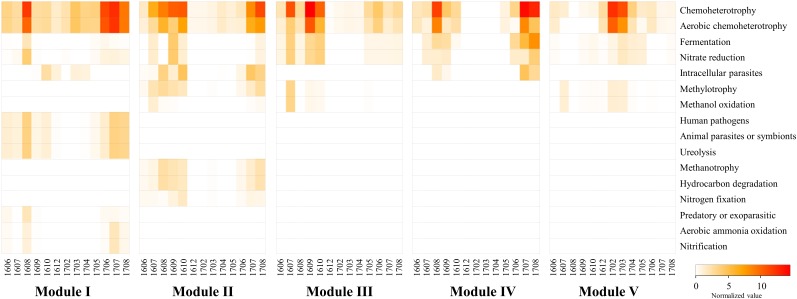
The heatmap showing the distribution of the functional groups of prokaryotic communities of modules using FAPROTAX. The normalized value was calculated by multiplying the calculated values (“function tables”) by the total sum of the OTUs belonging to each major module. Samples are labeled by a four-number code indicating the year and month of sampling (in YYMM format).

The analysis with FAPROTAX predicted the ecological and biological functions of the modules. A total of 16 microbial functional groups were identified for major modules using the FAPROTAX pipeline. Similar to the monthly pattern of the major modules, the functional structures of the non-cyanobacterial communities from major modules had significant differences amongst each other (PERMANOVA tests, pseudo-*F* = 3.27, *P* < 0.001) (Figure 7). The functional profiles of Module I, which increased with *Microcystis* blooms, were classified into human pathogens, animal parasites (or symbionts), and ureolysis that appeared during June-October 2016 and June-August 2017, while the functions related to aerobic ammonia oxidation, fermentation, and nitrate reduction increased during the *Microcystis* bloom peaks (i.e., August 2016 and July 2017) (Figure 7). Module IV, which increased with *Microcystis* blooms and lasted for 1 month after a bloom, was associated with increases in fermentation, nitrate reduction, and intracellular parasites during *Microcystis* bloom periods. In addition, the function related to fermentation and nitrate reduction increased in Module III before and after the *Microcystis* bloom peaks.

## Discussion

### Cyanobacterial Community Dynamics

During the sampling period, *Microcystis* generated serious blooms throughout the summer of 2017 (July–August) (Figure 2), while in 2016, it generated relatively temporary blooms (August). These results are in agreement with previous research (Christian et al., 1986) that suggests that even if water temperature and nutrient conditions are optimal for bloom formation, continuous bloom formation requires a stable water system (i.e., a low flow rate), which can be interrupted by a high flow rate caused by precipitation or discharge. The amount of precipitation and discharge in June–July 2016 was higher than that in 2017 (Supplementary Table S1). Interestingly, the relative abundance of *Pseudanabaena* increased during the *Microcystis* bloom period. These results are consistent with previous findings that *Pseudanabaena mucicola*, formerly referred to as *Phormidium mucicola*, could be epiphytic on *Microcystis* colonies and could co-occur during bloom periods (Vasconcelos et al., 2011; Agha et al., 2016). The mucilage of *Microcystis* colonies, which mainly consists of complex heteropolysaccharides, may provide favorable microenvironments for other cyanobacteria and non-cyanobacterial community members that would otherwise not survive in the prevailing environmental conditions during bloom periods in a eutrophic river (Brunberg, 1999). However, the mechanism of phycosphere formation in *Microcystis* is currently unclear. Our results revealed that approximately half of the bacteria that were directly linked to *Microcystis* or *Pseudanabaena* were overlapped, suggesting that these shared bacteria can play essential roles in the formation of *Microcystis*–*Pseudanabaena* phycosphere during bloom periods (Figure 6).

*Aphanizomenon* is usually reported in the summer, before and after *Microcystis* blooms, in the Nakdong River. However, serious blooms of *Aphanizomenon* have been observed in winter in the Nakdong River since 2015 (Park et al., 2018). We found that the December nCCCs were mainly influenced by the relative abundance of *Aphanizomenon* and by discharge (Figure 3). In addition, the high proportions of *Aphanizomenon* observed during the winter period could be explained by the massive multiplication of the genome and ribosomes in akinetes of *Aphanizomenon* (Sukenik et al., 2012). However, because of the lack of samples in November and January, no significant results were found in the MRAN during the winter season, and further studies are needed to determine the details of the relevant ecological mechanism.

### Non-cyanobacterial Community Dynamics

Throughout the sampling period, the nCCC had clear successional patterns in the Nakdong River. Multidimensional analysis revealed that temperature and discharge drove the nCCC succession. In addition to these abiotic factors, major cyanobacteria also contributed to reshaping the nCCC, especially during bloom periods. *Alcaligenaceae, Cytophagaceae*, OPB56, *Planctomycetaceae, Phycisphaeraceae*, and *Xanthomonadaceae* represented greater proportions of the nCCC during *Microcystis* bloom periods (Supplementary Figure S3). *Alcaligenaceae, Cytophagaceae*, and OPB56 were also reported as dominant bacterial groups during other *Microcystis* blooms (Yang et al., 2017). Certain groups of *Cytophagaceae* are cyanobacteria-lysing bacteria that are able to hydrolyse the cell walls of cyanobacteria and increase in abundance during bloom periods (Rashidan and Bird, 2001). Therefore, a *Microcystis* bloom might cause higher nutrient concentrations in the surrounding microenvironment by lysis-derived degradation, providing new energy sources for the abovementioned copiotrophic bacteria. Non-cyanobacterial community richness (Chao1) and diversity (Shannon’s index) generally increased in bloom peaks in both years (Supplementary Figure S5). It could be caused by the increase of non-cyanobacterial groups which were only associated with *Microcystis*. In addition, non-cyanobacterial community richness (Chao1) and amount of discharge were significantly correlated (Spearman rank order correlation, ρ = 0.58, *P* < 0.001). It suggested that a temporal increase of richness in September 2016 could be caused by a large amount of precipitation and discharge (Supplementary Table S1). Therefore, microbes from upper region could cause sudden increase in microbial richness.

### Network Analysis and CyanoHABs-Related Modules

Throughout the MRAN analysis, we found a complex network between the major cyanobacteria and nCCC, and this complex network had distinct modular attributes. The successional patterns of the major modules reflected the cyanoHABs development phases. Module I exactly followed the patterns of the *Microcystis* blooms, while Module IV showed similar patterns but maintained abundance until post-bloom periods (Figure 4D). Upon a closer examination of the components of these two modules, *Sporichthyaceae* (*Actinobacteria*) occupied approximately half of the bacterial community in Module IV. This group of bacteria has been reported to utilize cyanobacterial detritus as an energy source (Ghylin et al., 2014) and to form a large proportion of total bacteria in the post-bloom period in mesocosm and field studies (Berry et al., 2017; Cui et al., 2017). In contrast, *Comamonadaceae* and *Acetobacteraceae* were more abundant in Module I (Figure 4C). *Comamonadaceae* and *Acetobacteraceae* are known as specialists of nitrogen fixation (Saravanan et al., 2008; Pérez-Pantoja et al., 2012). During mass proliferation of *Microcystis*, a large flux of nitrogen sources were needed for cell growth. Therefore, these groups of bacteria could supply nitrogen source by nitrogen fixation to support high-density mats of *Microcystis*. Module III was negatively affected by the *Microcystis* bloom from July to September, while Module II was not negatively affected. *Saprospiraceae*, one of the major groups in Module III, is known as an algicidal bacterium that produces algicidal compounds that affect cyanobacteria and prey on cyanobacteria (Lewin, 1997). Taken together, a decrease in Module III could be a starting point for the formation of cyanoHABs. These fluctuations of cyanoHABs-related modules are likely important for understanding the formation, maintenance, and decomposition of cyanoHABs. Furthermore, the factors that control cyanoHABs should be considered from a modular microbial perspective rather than from the perspective of a single bacterial species.

Most of the OTUs directly linked with *Microcystis* were assigned to *Proteobacteria* (40.0%) and *Bacteroidetes* (32.0%) (Figure 6). Globally, these two groups, together with *Actinobacteria*, are frequently observed bacterial groups in cyanoHABs (Louati et al., 2015; Woodhouse et al., 2016, 2018; Xie et al., 2016). Importantly, several OTUs that were directly linked with *Microcystis* showed >99% similarity with those found in other aquatic ecosystems where *Microcystis* causes algal blooms. For instance, OTU00030 (OPB56), OTU00065 (*Methylophilus*), and OTU00158 (MWH-UniP1 group) were found in the free-living fraction in Villerest Lake in France during the *Microcystis* bloom period (Parveen et al., 2013), while OTU00057 (*Phenylobacterium*) and OTU00068 (*Paucimonas*) were found in the particle-associated fraction during cyanoHABs in Lake Tai in China (Cai et al., 2013; Shao et al., 2014). Furthermore, among these bacteria, positively correlated bacteria were included in Module I and Module IV, while negatively correlated bacteria were included in Module V (Supplementary Table S3). Therefore, we could conclude that the cyanoHABs-associated nCCC is conserved considerably across regions and could be a fundamental biological element for the formation and consolidation of cyanoHABs.

### Roles of Individual Species in the Modules

Because of the importance of modules in the microbial network structure, many studies have focused on identifying modules in networks and evaluating the roles of individual nodes in modules. From an ecological point of view, peripherals in a network might represent specialists, while module hubs/connectors are generalists, and network hubs act as supergeneralists (Olesen et al., 2007). Previous studies have indicated that the disappearance of module hubs and connectors might have serious impacts on the connection of modules in a global network structure (Guimera and Amaral, 2005; Olesen et al., 2007). Interestingly, one-fourth of the OTUs directly linked with cyanobacteria were also module hubs or connectors (Figure 5, 6). *Roseomonas* (OTU00041) has been reported to degrade an acyl homoserine lactone (AHL) (Chen et al., 2012). AHLs are one of the main types of quorum-sensing molecules characterized in bacteria. Previous research showed that *Microcystis* most likely produces AHLs (Zhai et al., 2012). Through the production and degradation of AHLs, the connector bacteria could modulate interactions between different modules in the whole microbial community network.

Module hubs are highly connected to many species within their own modules. Module hubs may represent the keystone species whose presence supports the establishment and growth of other taxa due to the desirable attribute the keystone species possess (Agler et al., 2016; Poudel et al., 2016). Agler et al. (2016) showed that hub microbes link host and abiotic factors to microbiome variation and have strong effects on epiphytic and endophytic bacterial colonization. Specifically, alpha diversity decreased and beta diversity stabilized in the presence of hub microbes. As mentioned above, our study found that nodes that belonged to *Dinghuibacter, Herbaspirillum, Cytophagaceae*, and the hgcI clade acted as module hubs in the network and were connected with major cyanobacteria. *Cytophagaceae* and *Herbaspirillum* are known as cyanobacterial growth-inhibiting (Rashidan and Bird, 2001) and growth-enhancing groups (Berg et al., 2009), respectively. Genus *Herbaspirillum* is known as plant-growth promoting diazotrophic bacterium that has ability to perform nitrogen fixation, phosphate solubilization, and siderophore production (Alves et al., 2015). In addition, *Herbaspirillum* produces indole acetic acid (IAA), a plant hormone of the auxin class (Bastián et al., 1998). Oliveira et al. (2009) discovered that this genus is potentially the most efficient species in the early colonization of plant roots. In this study, *Herbaspirillum* was assigned to module hub and occupied on average 0.25% in non-cyanobacterial community (maximum abundance: 5.36% during *Microcystis* bloom peak), suggesting that this module hub play a crucial role in primary colonization to phycosphere and stabilization of microbial diversity during bloom periods, similarly to root colonization in plants. These results suggested that bloom-related alterations of the nCCC could be caused by the cyanobacteria themselves (i.e., direct alteration) and by the connectors or module hubs (i.e., indirect alteration).

### Functional Annotation of CyanoHABs-Related Modules

Several techniques have been developed to predict the functional potential of microbial communities based on phylogeny (Langille et al., 2013; Louca et al., 2016). Functional prediction techniques could characterize the prokaryotic communities and provide general information and insights about ecosystem functions. However, there are two major limitations in applying this approach: (1) the basic presumption of FAPROTAX is that if the cultured members of a specific taxon can perform a particular function, then all members of the same taxon, both cultured and uncultured, can do that function; (2) the FAPROTAX database is not exhaustive; therefore, only small proportions of OTUs might be assigned to at least one functional group. Despite these limitations, the predicting putative functional groups using FAPROTAX is regarded as a useful alternative when metagenomic or metatranscriptomic data are not available. Interestingly, the functional profiles of the major modules were significantly different. In the cyanoHABs-related modules, functions related to fermentation, nitrate reduction, ureolysis, hydrocarbon degradation, and aerobic ammonia oxidation increased during *Microcystis* bloom periods compared to those during non-bloom periods, suggesting that these functions were related to the formation and decomposition of *Microcystis* (Figure 7). Freshwater ecosystems receive considerable amounts of urea from allochthonous sources such as runoff from land and sewage from urban areas. In addition, Siuda and Chrost (2006) reported that highest rates of urea hydrolysis were observed late in summer, during heavy phytoplankton blooms. Li et al. (2016) reported that the bioavailability of various nitrogen forms to *Microcystis* was in a decreasing order of urea, nitrate, nitrite, and ammonia. Therefore, the presence of cyanoHABs-related modules involved in the nitrogen cycle during the bloom period was presumed to contribute to the formation and maintenance of *Microcystis* blooms. *Cyanobacteria*, including *Microcystis*, possess the unique ability to naturally produce hydrocarbons (e.g., heptadecane, methylheptadecane, and ethyltetradecane) from fatty acids (Liu et al., 2013). Therefore, the increase in functions related to fermentation and hydrocarbon degradation in the cyanoHABs-related modules during bloom periods suggested that these bacterial modules were closely associated with the decomposition of *Microcystis* biomass and the degradation of *Microcystis*-derived substances (Shao et al., 2014).

In this study, the abundance of human pathogenic bacteria (e.g., *Roseomonas*) increased during *Microcystis* bloom periods compared to non-bloom periods. *Roseomonas* spp. are an important opportunistic pathogen and some species have been reported to cause serious infections in immunocompromised patients (Wang et al., 2012; Romano-Bertrand et al., 2016). In addition, Berg et al. (2009) isolated pathogenic bacteria from the water samples of cyanobacterial bloom. Therefore, pathogenic bacteria could be one of the reasons of adverse human health symptoms after contact to bloom water.

One of limitation of the study is that the samples were not differentially filtrated according to size. Several studies discovered dynamics and diversity of aggregate and free-living bacterial community during *Microcystis* bloom periods and they found that bacterial communities in aggregate and free-living fractions were different (Li et al., 2011; Shao et al., 2014; Yang et al., 2017). Size-fraction could provide more sophisticated interaction mechanisms. However, abundance of aggregate bacteria (particle fraction) could fluctuate in relation to abundance of particle materials, including mucilage of *Microcystis* and other filamentous organisms. Therefore, further study should be carried out to reveal dynamics of recurrent associations between aggregate and free-living bacterial community during Korean cyanoHABs together with results from analysis of total bacterial community.

This study focused on the microbial network in a eutrophic river during different cyanoHABs events. The nCCC showed significant changes during the cyanoHABs periods. The MRAN, a consensus-based association network, was developed to overcome some of the limitations of conventional network analysis and found strong correlations among cyanobacteria, non-cyanobacterial community members, and environmental variables. This approach revealed that fluctuations in cyanoHABs-related modules are a key factor in the formation, maintenance, and decomposition of cyanoHABs. Although only a few specific bacteria met the classification criteria of connectors and module hubs in distinct modules, one-fourth of the bacteria directly connected to bloom-forming cyanobacteria were module hubs and connectors. The potential functions of cyanoHABs-related modules during bloom periods included fermentation, nitrate reduction, nitrification, and aerobic ammonia oxidation. The ecological mechanisms behind cyanoHABs are still complex and dynamic. However, specific bacterial groups and modules contributed to different phases of cyanoHABs. Conversely, the local microbial network was found to be regulated by the types and phases of cyanoHABs. In conclusion, understanding the transition of microbial modular structures and the ecological roles of keystone species in relation to cyanoHABs could be two key points for understanding the mechanisms of cyanoHABs and controlling cyanoHABs.

## Data Availability

The datasets generated for this study can be found in Sequence Read Archive (SRA) of NCBI, the project number PRJNA479553.

## Author Contributions

S-JC analyzed the data, performed the experiments, and wrote the manuscript. YC and ARC performed the experiments. CSL, KB, and AC took samples and analyzed the environmental data. S-RK took samples. H-GL, H-MO, and C-YA revised the manuscript and designed the research. All authors reviewed and approved the manuscript.

## Conflict of Interest Statement

The authors declare that the research was conducted in the absence of any commercial or financial relationships that could be construed as a potential conflict of interest.

## Footnotes

1 http://www.mothur.org/wiki/MiSeq_SOP
