# Supplementary material for: Characterization of Distinct CyanoHABs-Related Modules in Microbial Recurrent Association Network
Source: Front Microbiol. 2019 Jul 17;10:1637. doi: 10.3389/fmicb.2019.01637 (PMC6650593; doi:10.3389/fmicb.2019.01637)
Supplement: Supplementary file 1 [file Data_Sheet_1.pdf]

Supplementary Material

Characterization of distinct cyanoHABs-related modules in microbial recurrent association network

Seong-Jun Chun[1, 2], Yingshun Cui[1], Chang Soo Lee[3], A Ra Cho[1], Kiwoon Baek[3], Ahyoung Choi[4], So-Ra Ko[1], Hyung-Gwan Lee[1], Seungwoo Hwang[5], Hee-Mock Oh[1, 2]** and Chi-Yong Ahn[1,2]*

[1]Cell Factory Research Center, Korea Research Institute of Bioscience and Biotechnology (KRIBB), Daejeon, Republic of Korea.

[2]Department of Environmental Biotechnology, KRIBB School of Biotechnology – Korea University of Science and Technology (UST), Daejeon, Republic of Korea.

[3]Division of Freshwater Bioresources Research, Nakdonggang National Institute of Biological Resources, Sangju, Republic of Korea.

[4]Division of Freshwater Bioresources Culture Research, Nakdonggang National Institute of Biological Resources, Sangju, Republic of Korea.

[5]Korean Bioinformation Center (KOBIC), Korea Research Institute of Bioscience and Biotechnology (KRIBB), Daejeon, Republic of Korea.

NUMBER OF TABLES: 3

NUMBER OF FIGURES: 5

* Corresponding author.

** Co-corresponding author.

Email addresses: cyahn@kribb.re.kr (C.-Y. Ahn), heemock@kribb.re.kr (H.-M. Oh).

Tel.: 82+42-860-4329; Fax: 82+42-860-4594

Table S1. Biophysicochemical characteristics (mean and standard deviation). Samples are labeled by a four-number code indicating the year and month of sampling (in YYMM format) and the numbers in parentheses indicate the number of samples per month.

Samples (n)	1606 (3)	1607 (3)	1608 (3)	1609 (6)	1610 (6)	1612 (6)	1702 (6)	1703 (6)	1704 (6)	1705 (6)	1706 (6)	1707 (6)	1708 (6)
Temperature (°C)	23.8±1	27.8±0.8	31.5±0.4	21.1±0.6	18.9±0.4	7.5±0.6	5.1±0.2	10.5±0.3	18.2±1	22.7±0.5	25.5±0.7	29.9±0.6	30±0.8
DO (mg/L)	12±3.2	12.1±1	16.4±2.1	6.5±0.2	9.3±0.2	11.3±1	14.2±6.1	11.4±0.3	9.9±0.8	8.4±1.1	9.9±0.4	9.7±1.3	8.6±2.4
pH	8.8±0.7	8.2±0.3	9.9±0.1	7.1±0.8	7.6±0.5	7.6±0.3	9.5±0.3	8.2±0.4	8.5±0.3	8.2±0.7	9.4±0.1	9.2±0.1	8.9±0.4
Chl-*a* (μg/L)	15±2.2	14.7±1	15.6±1.8	11.7±3	13.3±2.6	10.2±1.8	8.5±1.3	10.9±3.1	15±3	7.3±4.5	16.1±12.1	26.5±18.7	21.7±14.7
TN (mg/L)	1.9±0.2	2.7±0.2	2.6±0.7	2.5±0.3	2.5±0.1	2.4±0.1	2.9±0.3	2.8±0.7	2.5±0.5	2.8±0.5	1.6±0.1	1.7±0.3	3.1±0.4
TDN (mg/L)	1.7±0.1	2.5±0.3	1.9±0.3	2.3±0.3	2.2±0.2	2.1±0.2	2.7±0.3	2.2±0.8	2.4±0.3	2.5±0.2	1.4±0.1	1.5±0.3	2.8±0.3
TP (mg/L)	0.04±0	0.07±0.01	0.08±0.01	0.06±0.02	0.04±0	0.03±0	0.02±0	0.02±0.01	0.03±0.02	0.03±0.01	0.03±0.01	0.06±0.02	0.06±0.01
TDP (mg/L)	0.02±0.01	0.06±0.01	0.05±0.04	0.05±0.02	0.03±0.01	0.02±0	0.01±0	0.01±0	0.02±0.01	0.02±0.01	0.02±0.01	0.03±0.01	0.04±0.01
Discharge (m^3/s)	103±81	379±66	308±226	914±455	532±99	77±16	180±24	58±5	121±24	37±0	42±25	219±108	626±273
Monthly precipitation (mm)	115.2	188.3	141.5	407.7	182.4	103.8	33.8	35.7	105.1	39.2	49.8	172.1	82.5

Table S2. Distribution of significant correlations (edges) between phyla and environmental variables in the MRAN.

	Nodes	*Actinobacteria*	*Bacteroidetes*	*Planctomycetes*	*Proteobacteria*	*Verrucomicrobia*	Other bacteria	*Cyanobacteria*	Environmental variables
Actinobacteria	39	**90 (741, 12.1%)**							
Bacteroidetes	89	61 (3471, 1.8%)	**197 (3916, 5%)**						
Planctomycetes	33	57 (1287, 4.4%)	63 (2937, 2.1%)	**80 (528, 15.2%)**					
Proteobacteria	117	204 (4563, 4.5%)	260 (10413, 2.5%)	152 (3861, 3.9%)	**474 (6786, 7%)**				
Verrucomicrobia	38	60 (1482, 4%)	49 (3382, 1.4%)	41 (1254, 3.3%)	147 (4446, 3.3%)	**97 (703, 13.8%)**			
Other bacteria	23	38 (897, 4.2%)	48 (2047, 2.3%)	35 (759, 4.6%)	108 (2691, 4%)	25 (874, 2.9%)	**36 (253, 14.2%)**		
Cyanobacteria	16	**34 (624, 5.4%)**	27 (1424, 1.9%)	22 (528, 4.2%)	74 (1872, 4%)	20 (608, 3.3%)	12 (368, 3.3%)	**40 (120, 33.3%)**	
Environmental variables	7	10 (273, 3.7%)	9 (623, 1.4%)	3 (231, 1.3%)	23 (819, 2.8%)	6 (266, 2.3%)	4 (161, 2.5%)	3 (112, 2.7%)	**11 (21, 52.4%)**

'Nodes' indicates the number of OTUs or environmental variables in the MRAN; the remaining columns indicate" the number of significant pairwise correlations and in parentheses, the total number of possible correlations and the ratio of significant correlations/possible correlations between node types. Data for crosses with ratios of significant correlations/possible correlations above 5% are marked in bold.

Table S3. Characteristics of the non-cyanobacterial OTUs that correlate with cyanobacterial OTUs in the MRAN.

OTUs	Role	Int.	Rel. abund. (aver., max.)	Module	Taxonomical identification
**Correlated with Otu00002 (*Microcystis*)**					
Otu00087		pc	0.23, 0.94	I	*Actinobacteria*; *Actinobacteria*; *Frankiales*; *Sporichthyaceae*; hgcI_clade
Otu00207		nc	0.11, 0.48	I	*Actinobacteria*; *Actinobacteria*; *Frankiales*; *Sporichthyaceae*; hgcI_clade
Otu00027		pc	0.44, 13.7	I	*Proteobacteria*; *Betaproteobacteria*; *Burkholderiales*; *Alcaligenaceae*; uncultured
Otu00123		nc	0.16, 1.13	V	*Proteobacteria*; *Betaproteobacteria*; *Burkholderiales*; *Comamonadaceae*; *Limnohabitans*
Otu00043		nc	0.44, 4.98	V	*Flavobacteriia*; *Flavobacteriales*; *Flavobacteriaceae*; *Flavobacterium*
Otu00051		nc	0.3, 3.06	V	*Verrucomicrobia*; *Opitutae*; *Opitutales*; *Opitutaceae*; *Opitutus*
Otu00084		pc	0.17, 1.99	I	*Verrucomicrobia*; *Opitutae*; *Opitutales*; *Opitutaceae*; *Opitutus*
Otu00060	Connector	pc	0.25, 1.55	IV	*Planctomycetes*; *Phycisphaerae*; *Phycisphaerales*; *Phycisphaeraceae*; CL500-3
Otu00038		nc	0.42, 3.29	V	*Planctomycetes*; *Planctomycetacia*; *Planctomycetales*; *Planctomycetaceae*; uncultured
Otu00145	Module hub	nc	0.12, 0.95	V	*Bacteroidetes*; *Sphingobacteriia*; *Sphingobacteriales*; *Chitinophagaceae*; *Dinghuibacter*
Otu00160		pc	0.11, 1.73	I	*Bacteroidetes*; *Sphingobacteriia*; *Sphingobacteriales*; *Chitinophagaceae*; *Sediminibacterium*
Otu00122		pc	0.11, 1.14	I	*Bacteroidetes*; *Sphingobacteriia*; *Sphingobacteriales*; *LiUU*-11-161; uncultured
Otu00089		pc	0.19, 1.99	I	*Bacteroidetes*; *Sphingobacteriia*; *Sphingobacteriales*; *Saprospiraceae*; uncultured
Otu00282		ndl	0.14, 1.59	V	*Bacteroidetes*; *Sphingobacteriia*; *Sphingobacteriales*; *Sphingobacteriaceae*; *Pedobacter*
Otu00031		nc	0.42, 2.65	V	*Bacteroidetes*; *Sphingobacteriia*; *Sphingobacteriales*; *Sphingobacteriaceae*; *Solitalea*
**Correlated with Otu00008 (*Pseudanabaena*)**					
Otu00118		pc	0.13, 1.62	IV	*Actinobacteria*; *Actinobacteria*; *Micrococcales*; *Microbacteriaceae*; Candidatus_*Aquiluna*
Otu00069		nc	0.37, 2.01	I	*Proteobacteria*; *Betaproteobacteria*; *Burkholderiales*; *Burkholderiaceae*; *Polynucleobacter*
Otu00025		nc	2.5, 35.93	I	*Proteobacteria*; *Betaproteobacteria*; *Burkholderiales*; *Comamonadaceae*; *Limnohabitans*
Otu00044	Connector	nc	0.38, 3.26	V	*Proteobacteria*; *Betaproteobacteria*; *Burkholderiales*; *Comamonadaceae*; *Polaromonas*
Otu00384		pc	0.04, 0.28	I	*Proteobacteria*; *Betaproteobacteria*; *Burkholderiales*; *Comamonadaceae*; unclassified
Otu00061	Module hub	pc	0.25, 5.36	I	*Proteobacteria*; *Betaproteobacteria*; *Burkholderiales*; *Oxalobacteraceae*; *Herbaspirillum*
Otu00110		pc	0.14, 2.55	I	*Chlorobi*; *Chlorobia*; *Chlorobiales*; OPB56; uncultured
Otu00047		nc	0.32, 2.47	I	*Bacteroidetes*; *Flavobacteriia*; *Flavobacteriales*; *Cryomorphaceae*; *Fluviicola*
Otu00039		pc	0.28, 3.95	I	*Bacteroidetes*; *Flavobacteriia*; *Flavobacteriales*; *Flavobacteriaceae*; *Flavobacterium*
Otu00253		pc	0.07, 0.66	IV	*Proteobacteria*; *Gammaproteobacteria*; *Xanthomonadales*; *Xanthomonadales*_Incertae_Sedis; *Acidibacter*
Otu00112		pc	0.17, 2.79	IV	*Planctomycetes*; *Planctomycetacia*; *Planctomycetales*; *Planctomycetaceae*; *Pirellula*
Otu00141		pc	0.12, 1.09	IV	*Bacteroidetes*; *Sphingobacteriia*; *Sphingobacteriales*; *Chitinophagaceae*; *Terrimonas*

Table S3. continued

**Correlated with Otu00002 (*Microcystis*) and Otu00008 (*Pseudanabaena*)**					
Otu00057		pc	0.28, 4.97	I	*Proteobacteria*; *Alphaproteobacteria*; *Caulobacterales*; *Caulobacteraceae*; *Phenylobacterium*
Otu00138	Connector	pc	0.12, 1.1	I	*Proteobacteria*; *Alphaproteobacteria*; *Rhodobacterales*; *Rhodobacteraceae*; *Rhodobacter*
Otu00041	Connector	pc	0.39, 1.42	I	*Proteobacteria*; *Alphaproteobacteria*; *Rhodospirillales*; *Acetobacteraceae*; *Roseomonas*
Otu00067		pc	0.27, 2.94	I	*Proteobacteria*; *Alphaproteobacteria*; *Rhodospirillales*; *Acetobacteraceae*; *Roseomonas*
Otu00158	Connector	pdr, pc	0.14, 1.5	IV	*Proteobacteria*; *Betaproteobacteria*; *Burkholderiales*; *Alcaligenaceae*; MWH-UniP1_aquatic_group
Otu00114		nc	0.16, 1.69	V	*Proteobacteria*; *Betaproteobacteria*; *Burkholderiales*; *Comamonadaceae*; *Albidiferax*
Otu00068		pc	0.28, 1.27	I	*Proteobacteria*; *Betaproteobacteria*; *Burkholderiales*; *Oxalobacteraceae*; *Paucimonas*
Otu00065		nc	0.31, 2.03	V	*Proteobacteria*; *Betaproteobacteria*; *Methylophilales*; *Methylophilaceae*; unclassified
Otu00030		pc	0.4, 2.75	I	*Chlorobi*; *Chlorobia*; *Chlorobiales*; OPB56; uncultured
Otu00050	Module hub	pc	0.28, 4.3	I	*Bacteroidetes*; *Cytophagia*; *Cytophagales*; *Cytophagaceae*; uncultured
**Correlated with Otu00035 (*Dolichospermum*)**					
Otu00033	Connector	pc	0.69, 4.12	II	*Verrucomicrobia*; *Spartobacteria*; *Chthoniobacterales*; *Chthoniobacterales*_Incertae_Sedis; *Terrimicrobium*
Otu00072		pc	0.3, 1.55	II	*Proteobacteria*; *Betaproteobacteria*; *Burkholderiales*; *Burkholderiaceae*; *Limnobacter*
Otu00074	Connector	pc	0.27, 1.02	III	*Proteobacteria*; *Gammaproteobacteria*; *Xanthomonadales*; *Xanthomonadales*_Incertae_Sedis; *Acidibacter*
Otu00097	Module hub	pc	0.24, 1.11	II	*Actinobacteria*; *Frankiales*; *Sporichthyaceae*; hgcI_clade
Otu00143		pc	0.15, 0.95	II	*Proteobacteria*; *Betaproteobacteria*; *Burkholderiales*; *Comamonadaceae*; *Acidovorax*
Otu00263		pc	0.06, 1.37	II	*Bacteroidetes*; *Flavobacteriia*; *Flavobacteriales*; *Flavobacteriaceae*; *Flavobacterium*
Otu00264		pc	0.07, 0.43	II	*Proteobacteria*; *Gammaproteobacteria*; *Xanthomonadales*; *Xanthomonadales*_Incertae_Sedis; *Acidibacter*
**Correlated with Otu00013 (*Dolichospermum*)**					
Otu00058		pc	0.32, 1.89	V	*Bacteroidetes*; *Sphingobacteriia*; *Sphingobacteriales*; *Chitinophagaceae*; *Sediminibacterium*

'Int.' indicates the type of interaction; 'pc' and 'nc' represent positive and negative correlation, respectively; 'ndl' and 'pdr' represent negative- (left) and positive-delayed (right) correlations, respectively. 'Rel. abund.' indicates average abundance of each OTU with maximum relative abundance (%)in parentheses.

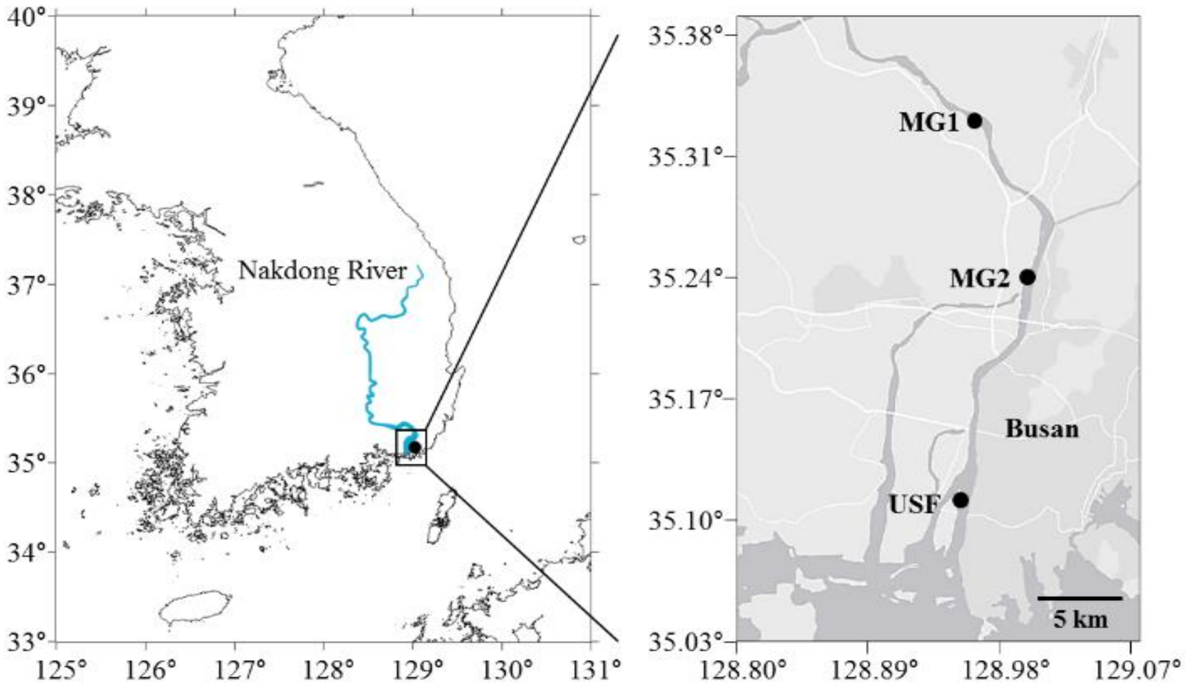

Fig. S1. Map of water sample collection sites in the Nakdong River (MG1: 35.33°N, 128.96°E, MG2: 35.24°N, 129.00°E, and USF: 35.11°N, 128.95°E).

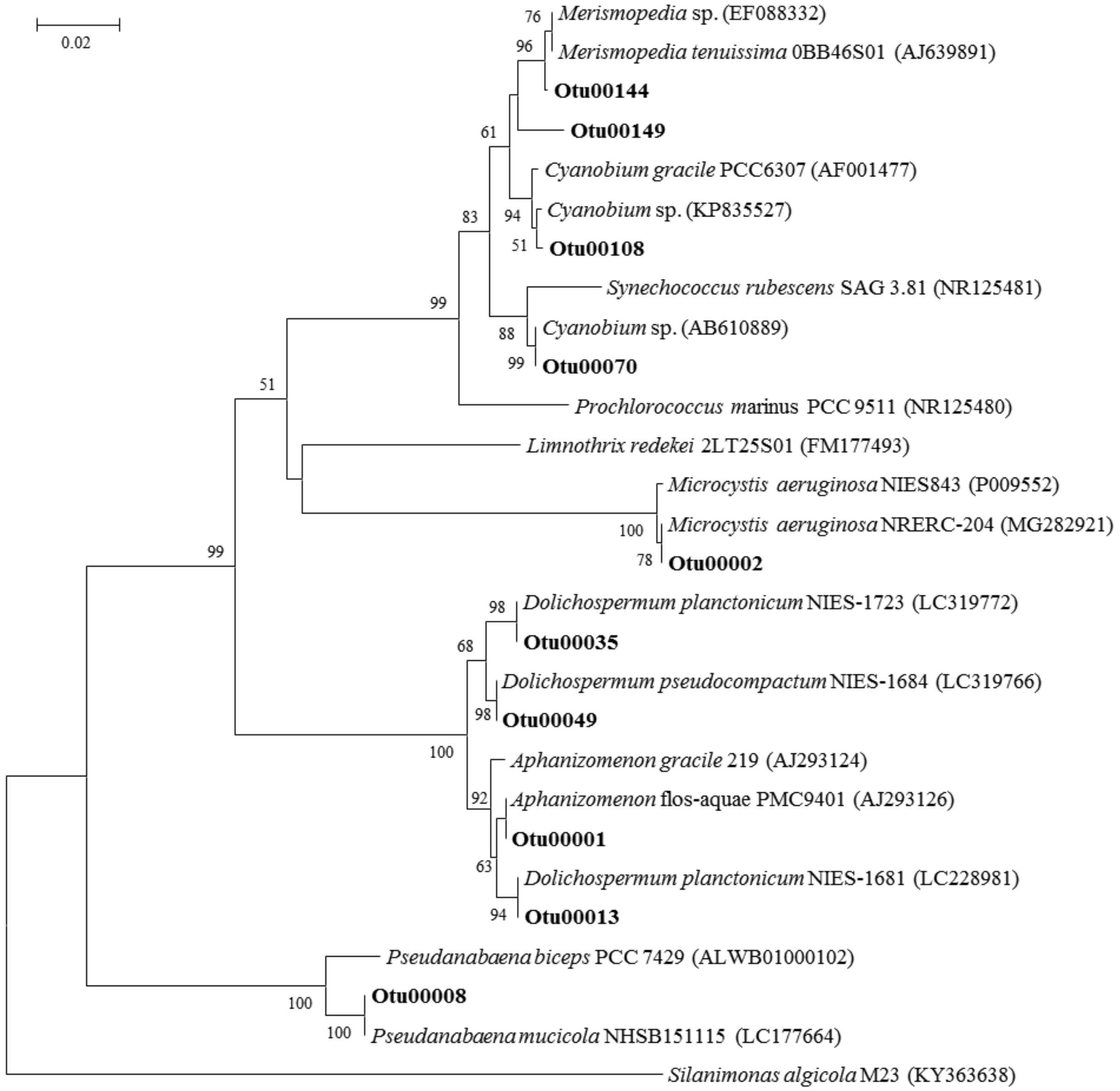

Fig. S2. Phylogenetic dendrogram of neighbor-joining clusters of the major cyanobacterial OTUs. Numbers at the nodes represent bootstrap values (%) from the neighbor-joining algorithm. Only bootstrap values above 50% are shown. GenBank accession numbers for the sequences are shown in parentheses.

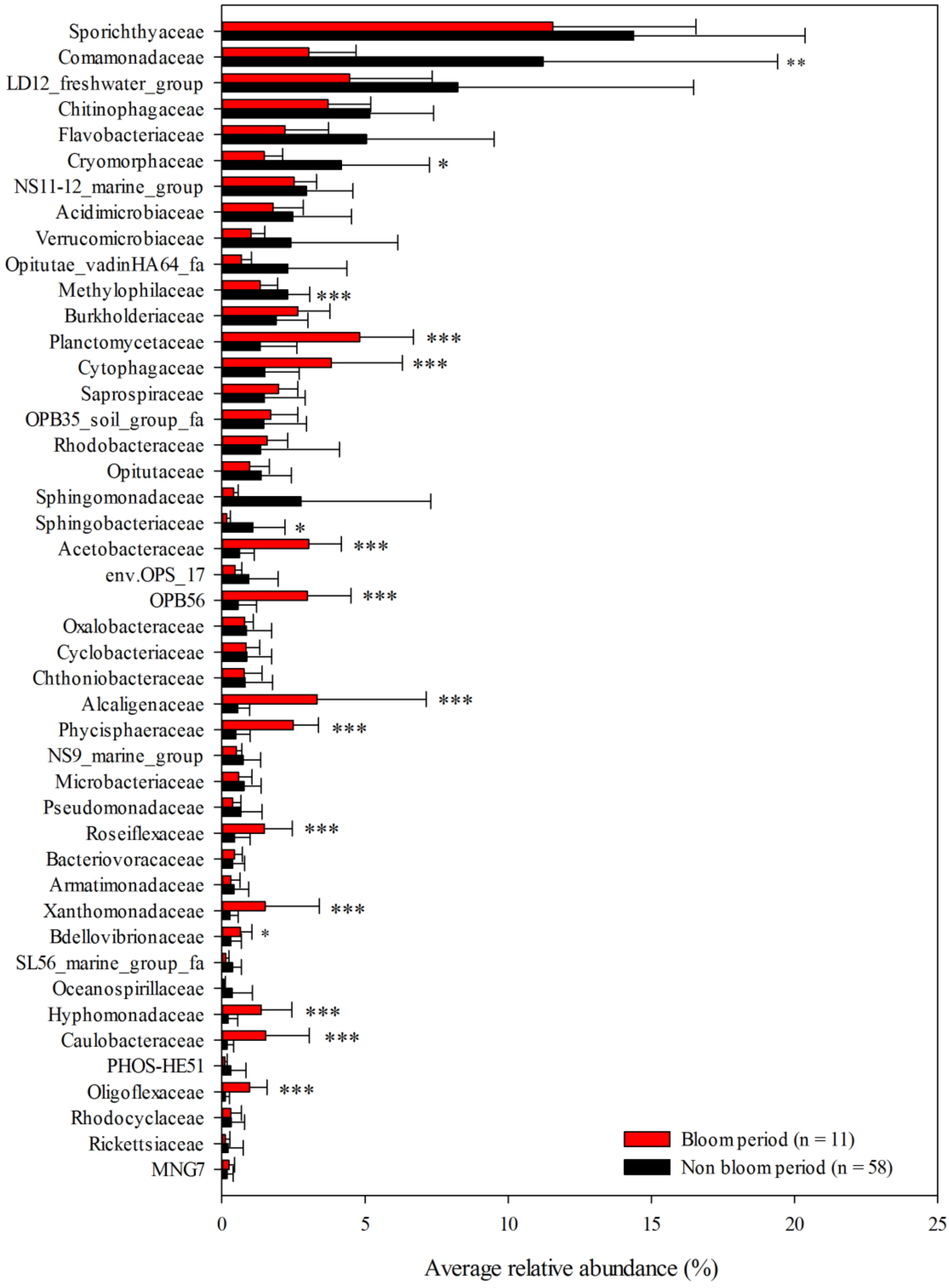

Fig. S3. Relative abundances of non-cyanobacterial OTUs (family level). $P < 0.01$ *. $P < 0.005$ **. $P < 0.001$ ***.

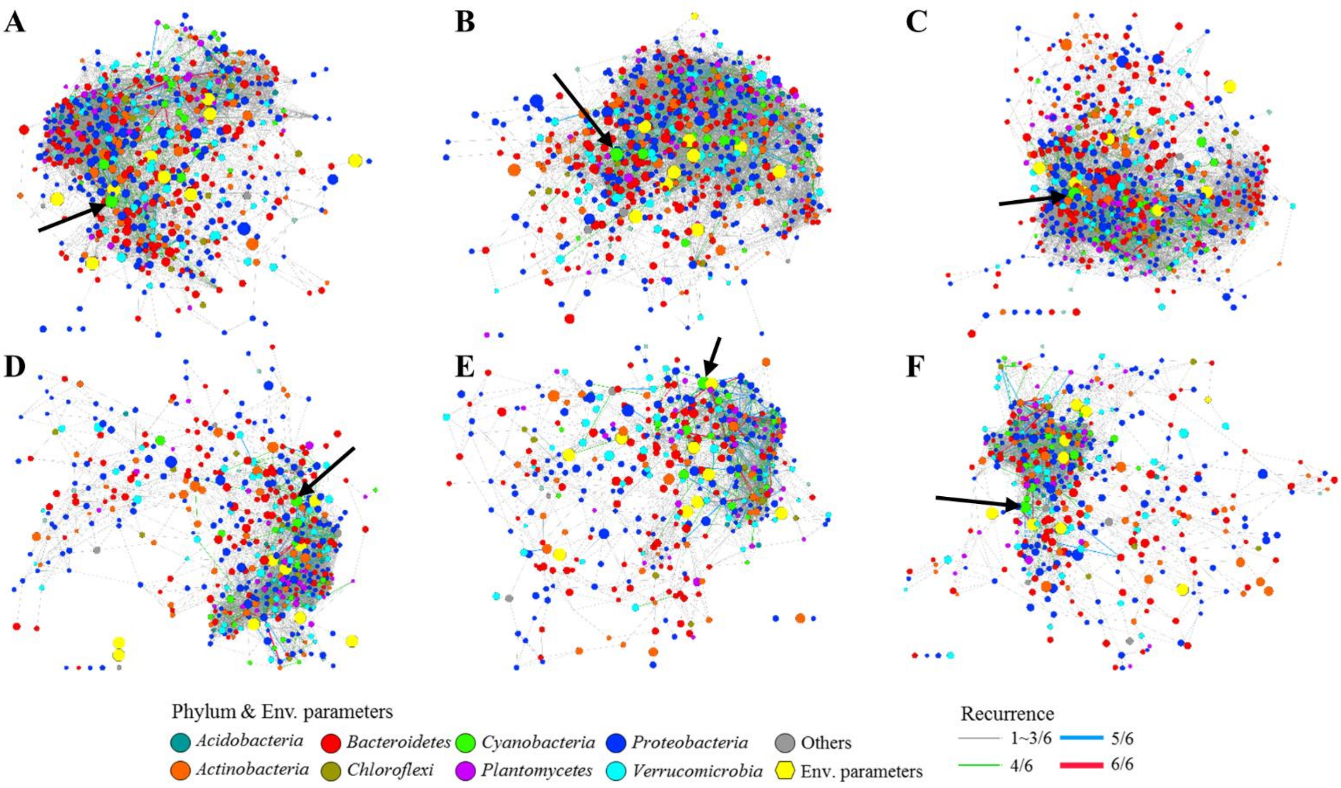

Fig. S4. Association network analysis of the bacterial community at each site and depth. (A) MG1_0 m, (B) MG2_0 m, (C) USF_0 m, (D) MG1_2 m, (E) MG2_2 m, (F) USF_2 m. Black arrows indicate *Microcystis* (OTU00002).

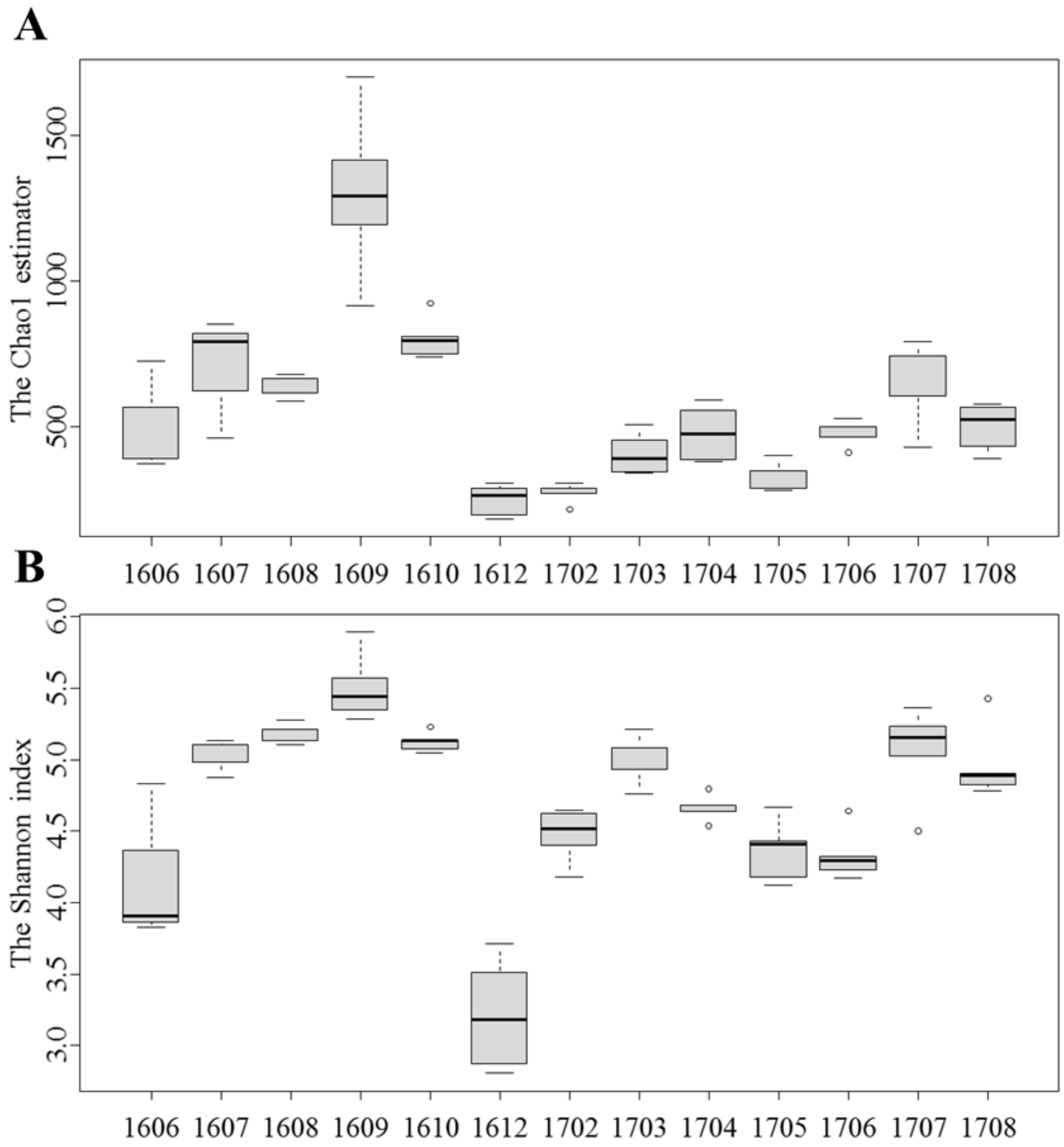

Fig. S5. Alpha diversity indices of heterotrophic bacterial communities. (A) richness, (B) diversity. Data are expressed as standard boxplots with medians. Outliers are shown as dots. Samples are labeled by a four-number code indicating the year and month of sampling (in YYMM format).
